# Repolarization of the action potential enabled by Na^+ ^channel deactivation in PSpice simulation of cardiac muscle propagation

**DOI:** 10.1186/1742-4682-2-48

**Published:** 2005-12-12

**Authors:** Lakshminarayanan Ramasamy, Nicholas Sperelakis

**Affiliations:** 1 Dept. of Electrical Computer Engineering and Computer Science, University of Cincinnati College of Engineering, Cincinnati, OH 45219, USA; 2 Dept. of Molecular & Cellular Physiology, University of Cincinnati College of Medicine, Cincinnati, OH 45267-0576, USA

## Abstract

**Background:**

In previous studies on propagation of simulated action potentials (APs) in cardiac muscle using PSpice modeling, we reported that a second black-box (BB) could not be inserted into the K^+ ^leg of the basic membrane unit because that caused the PSpice program to become very unstable. Therefore, only the rising phase of the APs could be simulated. This restriction was acceptable since only the mechanism of transmission of excitation from one cell to the next was being investigated.

**Methods and results:**

We have now been able to repolarize the AP by inserting a second BB into the Na^+ ^leg of the basic units. This second BB effectively mimicked deactivation of the Na^+ ^channel conductance. This produced repolarization of the AP, not by activation of K^+ ^conductance, but by deactivation of the Na^+ ^conductance. The propagation of complete APs was studied in a chain (strand) of 10 cardiac muscle cells, in which various numbers of gap-junction (gj) channels (assumed to be 100 pS each) were inserted across the cell junctions. The shunt resistance across the junctions produced by the gj-channels (R_gj_) was varied from 100,000 M? (0 gj-channels) to 10,000 M? (1 gj-channel), to 1,000 M? (10 channels), to 100 M? (100 channels), and 10 M? (1000 channels). The velocity of propagation (θ, in cm/s) was calculated from the measured total propagation time (TPT, the time difference between when the AP rising phase of the first cell and the last cell crossed -20 mV, assuming a cell length of 150 μm. When there were no gj-channels, or only a few, the transmission of excitation between cells was produced by the electric field (EF), i.e. the negative junctional cleft potential, that is generated in the narrow junctional clefts (e.g. 100 A) when the prejunctional membrane fires an AP. When there were many gj-channels (e.g. 1000 or 10,000), the transmission of excitation was produced by local-circuit current flow from one cell to the next through the gj-channels.

**Conclusion:**

We have now been able to simulate complete APs in cardiac muscle cells that could propagate along a single chain of 10 cells, even when there were no gj-channels between the cells.

## Background

There are no low-resistance connections between the cells in several different cardiac muscle and smooth muscle preparations [1-3]. In a computer simulation study of propagation in cardiac muscle, it was shown that the electric field (EF) generated in the narrow junctional clefts when the prejunctional membrane fires an action potential (AP) depolarizes the postjunctional membrane to its threshold [4-7]. This results in excitation of the postjunctional cell after a brief junctional delay. The total propagation time consists primarily of the summed junctional delays. This results in a staircase-shaped propagation, the surface sarcolemma of each cell firing almost simultaneously [5]. Propagation has been shown to be discontinuous (or saltatory) in cardiac muscle [8-11]. Fast Na^+ ^channels are localized in the junctional membranes of the intercalated disks in cardiac muscle [6,12,13], a requirement for the EF mechanism to work [4,5]. Propagation in the heart still occurs in connexin-43 and Cx40 knockout mice, but it is slowed [14-17], as predicted by our PSpice simulation study.

We have modeled APs in cardiac muscle using the PSpice program for circuit design and analysis, and we have corroborated our earlier reports that the EF developed in the junctional cleft is sufficiently large to allow the transfer of excitation without the requirement for a gap junction [7,18,19]. In those studies, we found that a second black-box (BB-2) could not be inserted into the K^+ ^leg of the Hodgkin-Huxley circuit of the basic membrane units because that caused the PSpice program to become very erratic and unstable. Therefore, only the rising phase of the APs was simulated. This defect in the program was acceptable since only the mechanism(s) for the transfer of excitation from one cell to the next was under investigation; for this purpose, only the fast rising phase of the AP was necessary. The purpose of the present study was to determine whether another method for repolarization could be devised that would circumvent the instability problem. We inserted a second BB (BB-2) into the same leg as the first (BB-1), i.e. the Na^+ ^leg, to mimic the deactivation of the Na^+^channel and thereby produce repolarization of the AP. This method worked, as it did in the case of smooth muscle and the Ca^++ ^channel [18]. Thus, repolarization was produced not by activation of the K^+ ^conductance, but instead by deactivation of the Na^+ ^conductance.

## Methods

Details of the methods and circuit parameters used for cardiac muscle were described previously [3,7]. There were two surface membrane units in each cell (one facing upwards and one inverted) and one unit for each of the junctional membranes. The values for the circuit parameters used (standard conditions) are listed in Table 1. The cardiac muscle cell was assumed to be a cylinder 150 μm long and 16 μm in diameter. The cell input resistance was assumed to be 20 M?. A junctional tortuosity (interdigitation) factor of 4 was assumed for the cell junction [3]. The junctional cleft potential (V_jc_) is produced across R_jc_, the radial resistance of the narrow and tortuous junctional cleft. The junctional cleft contained two radial resistances (R_jc_) of 40 M? each in parallel (i.e. R_jc _= 20 M?). The value assigned to R_jc _reflects the thickness of the junctional gap (end-to-end) and the tortuosity factor.

**Table 1:** Parameter values used under standard conditions.

**Parameters**	**Surface unit **	**Junctional Unit**
C_m_(fF)	300	30
R_K_(MΩ)	71	710
R_Na_(MΩ)	710	7100
E_K_(mV)	-94	-94
E_Na_(mV)	+60	+60
R_d_(MΩ)	5000	5000
C_d_(pF)	30	30
	**Common**	
R_or_(KΩ)	1.0	
R_ol_(KΩ)	1.0	
Ri (KΩ)	100	
R_jc_(MΩ)	20 (40 / 2)	
R_BT_(KΩ)	1.0	

The circuit used for each unit was kept as simple as possible, using only those ion channels that set the resting potential (RP) and predominate during the rising phase of the AP. The RP was -80 mV and the overshoot potential was +30 mV (AP amplitude of 110 mV). Because the PSpice program does not have a V-dependent resistance to represent the increase in conductance for Na^+ ^ions during excitation, this function was simulated by a V-controlled current source (our "black-box", BB) in each of the basic circuit units. The current output of the BB at various membrane voltages was calculated assuming a sigmoidal relationship between membrane voltage and resistance.

**Figure 1:**
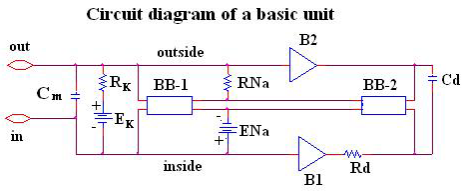
Circuit diagram for one of the basic units. The unit circuitry was the same for both the surface units and junctional units, but the values of the various components were adjusted to reflect the long surface membrane and short junctional membrane. These values are listed in Table 1. The GTABLE values for the two types of membrane were also different, and these are listed in Table 2. Note that there are two black-boxes (BB) in the basic unit, and both are in the same leg of the Hodgkin-Huxley circuit, namely the Na^+ ^conductance leg. The first BB produced activation of the Na^+ ^conductance, and the second produced deactivation of the Na^+ ^channel conductance. It was necessary to produce a time delay (RC time constant) before the second BB came into play to cause deactivation. The two operational amplifiers (unit gain) acted as buffers. In the K^+ ^leg, the K^+ ^conductance is in series with E_K_, the K^+^equilibrium potential.

**Figure 2:**
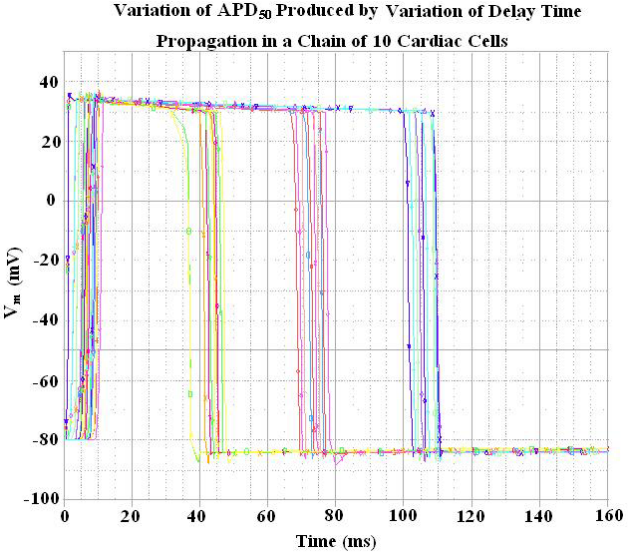
Records showing how variations in the delay time (RC time constant), controlling the activation of the second black box (BB-2), affected the duration of the action potentials (APD_50_). The records were obtained with propagation in a chain of 10 cells. Shortening the delay time produced shortening of the APD_50_.

Our novel approach to simulation of the entire action potential (AP) waveform was achieved by inserting a second BB into the sodium leg (Na^+^) of the basic unit. Thus, the first BB mimics Na^+ ^activation, and the second BB mimics deactivation of the Na^+^-channel conductance. The latter allowed repolarization of the AP to occur. BB-2 is connected between the outside and inside of the membrane unit, with reversed polarity compared to BB-1, as shown in Figure 1. The outputs of BB-1 and BB-2 were tied together in such a way that BB-2's output current nullifies BB-1's output current. BB-2 was activated with a delay time corresponding to the physiological delay value (i.e. to give an appropriate APD_50_). The required delay time was generated using a delay element R_d, _C_d,_(RC time constant), as shown in Figure 1. Figure 2 illustrates how the duration of the cardiac action potentials (APD_50_) in the chain of 10 cells can be varied over a wide range by changing the RC time constant. As can be seen, decreasing the delay time results in shortening of the AP.

At the resting potential, BB-2's output current is set to 0 nA. Once the cell has fired using BB-1's current, the potential across the input of BB-2 starts increasing with a rate corresponding to the RC time constant of the delay element. Thereby, BB-2 starts responding to the rising input voltage. Two buffer elements (unity gain operational amplifiers) were added to isolate the input terminal of BB-2 from BB-1. Thus, any hindrance of BB1's function by BB-2 was avoided.

The cells in the chain were either connected by low-resistance pathways (gap-junction channels) or not interconnected, so that transmission of excitation from one cell to the next had to be by the electric field (EF) developed in the narrow junctional cleft. The ends of the chain had a bundle termination resistance (R_BT_) of 1.0 K? to mimic the physiological condition. Electrical stimulation (rectangular current pulses of 0.25 nA and 0.50 ms duration) was applied to the inside of the first cell of the chain (cell #1). To minimize confusion, the voltage was recorded from only one surface unit (upward-facing) in each cell. Propagation velocity was calculated from the total propagation time (TPT), measured as the difference between the times when the APs (rising phase) of the first cell and that of the last cell crossed -20 mV; the cell length was assumed to be 150 μm.

## Results

**Figure 3:**
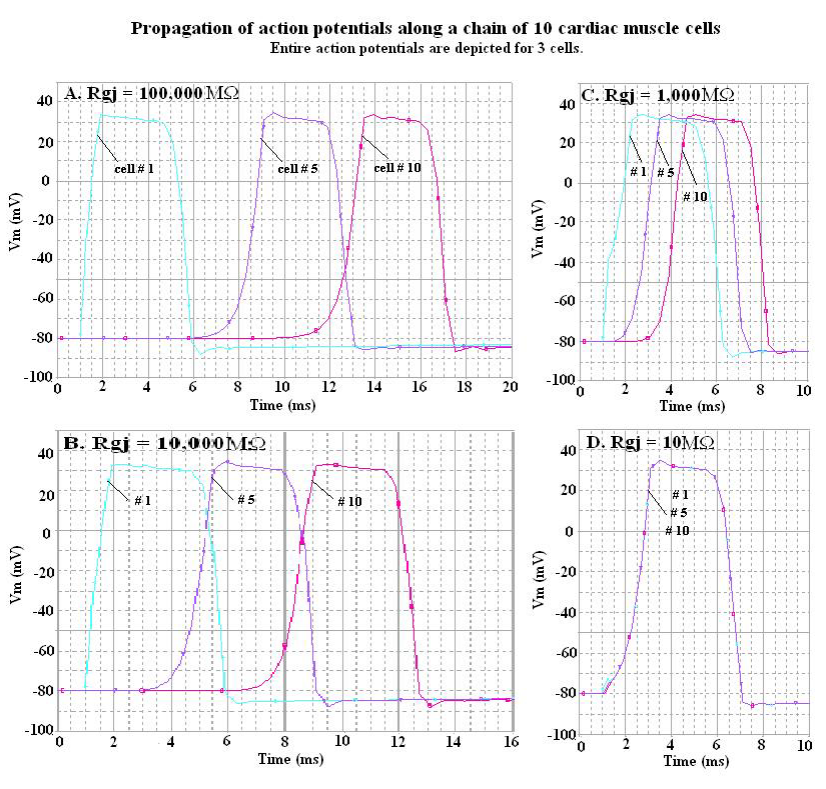
Propagation of APs simulated by PSpice along a chain of ten cardiac muscle cells. The entire AP is depicted, but to avoid confusion the records illustrate only three of the cells: cells # 1, 5 and 10. The four panels show the effect of varying the number of gj-channels from zero (panel A, R_gj _= 100,000 MΩ), to one (panel B, R_gj _= 10,000 MΩ), to 10 (panel C, R_gj _= 1,000 MΩ), to 1000 (panel D, R_gj _= 10 MΩ). When there were many gj-channels (D), the APs of all 10 cells were superimposed, indicating extremely fast propagation velocity.

The AP waveform and propagation down the chain of 10 cells is illustrated in Figure 3. To avoid confusion, records are illustrated for only cells 1, 5 and 10. Propagation was studied with various numbers of gj-channels traversing the cell junctions, namely 0, 1, 10, 100 and 1,000. Assuming each gj-channel has a conductance of 100 pS, these channels corresponded to shunt resistances across each junction (R_gj_) of 100,000 M?, 10,000 M?, 1,000 M? and 100 M?, respectively. The corresponding records are shown in panels A, B, C and D of Figure 3.

When there were no gj-channels (**A**), or only one channel (**B**), fast propagation still occurred, which was initiated by the EF mechanism. When there are many gj-channels (e.g., 1000 (**D**)), then the APs of all 10 cells are superimposed. Thus, the TPT (measured at a V_m_of -20 mV) is that of a single AP, which is approximately 0.004 ms in panel D. Hence, the propagation velocity (θ) appears to be nearly infinite.

**Figure 4:**
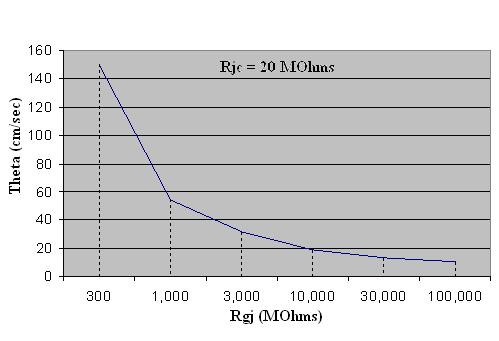
Graphic summary of the propagation velocity (θ) as a function of the shunt resistance across the 9 cell junctions (R_gj_). TPT was the difference between the times when the AP of cell #1 and cell #10 crossed a V_m _of -20 mV. The velocity (θ) was calculated from the TPT assuming a cell length of 150 μm. Assuming the conductance of the gj-channel is 100 pS, the R_gj _values of 10, 100, 1,000, 10,000 and 100,000 MΩ correspond to the number of gj-channels of 1,000, 100, 10, 1.0 and 0. For the purpose of graphic presentation, runs were also made for Rgj values of 30, 300, 3,000 and 30,000.

Figure 4 shows a plot of θ as a function of R_gj. _As R_gj _decreases (reflecting more and more gj-channels), θ increases. These data are also summarized in Table 3 to facilitate quantitative comparison.

**Figure 5:**
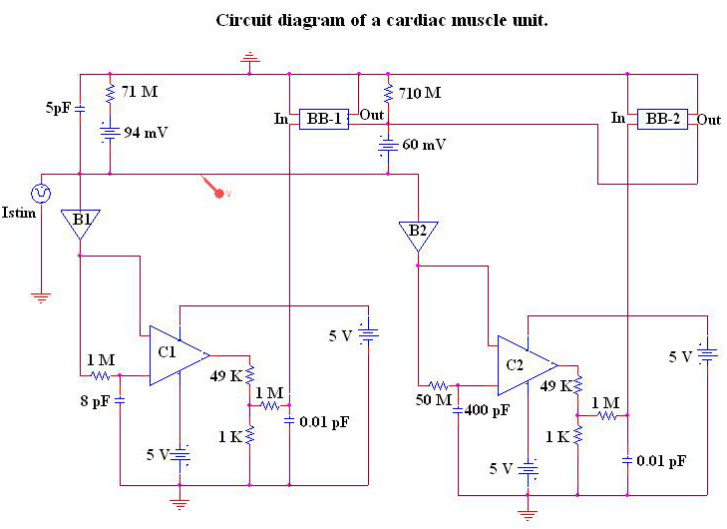
Diagram of the circuit used to show a more accurately modeled cardiac action potential with spike and plateau components.

**Figure 6:**
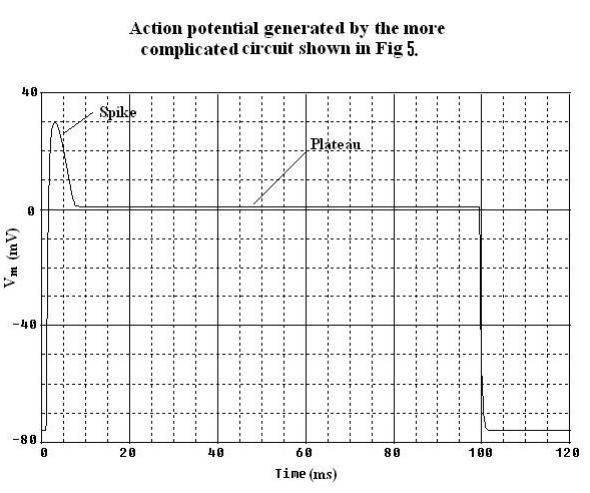
Action potential generated by the more complex circuit given in Fig 5. It was possible with this circuit to generate an AP waveform for cardiac muscle cells with spike and plateau components. This figure should be compared with Fig 2.

## Discussion

The present results demonstrate that we can simulate the entire cardiac AP, both the rising phase and the falling phase, using PSpice. Since a second BB (BB-2) could not be inserted into the K^+ ^leg of the basic Hodgkin-Huxley membrane units because of instability in the PSpice program, the problem was solved by inserting the BB-2 into the Na^+ ^leg to produce deactivation of the Na^+ ^conductance turned on by the first BB (BB-1). BB-2 supplied current in the opposite polarity to that supplied by BB-1, essentially canceling it out and giving a net current of zero. BB-2 supplied its current after a time delay provided by an RC time constant. Thus, BB-1 produced the rising phase of the AP and BB-2 produced the falling (repolarizing) phase. Therefore, it is now possible to use the PSpice program to study propagation of complete APs in single strands and in two-dimensional sheets (parallel strands).

When there were no low-resistance connections or gj-channels between the cells, propagation was still rapid because of the EF mechanism. When there were many gj-channels (e.g. 1000), the propagation velocity became extremely fast, i.e. non-physiological.

The after-hyperpolarization produced following the polarization of the AP (e.g. see Fig. 2), equivalent to the physiological hyperpolarizing after-potential seen in some excitable cells (e.g. smooth muscle cells, cardiac pacemaker cells and neurons), is due to a "feed-forward" mechanism. When BB-2 is supplying its opposing current and producing partial repolarization of the AP, the current supplied by BB-1 starts to decrease because the BB-GTABLE is reversible [19]. Therefore, BB-2 supplies an excess of current, causing the after-hyperpolarization.

The PSpice modeling techniques can be used to study propagation of excitation in various tissues including cardiac muscle, smooth muscle and neurons. The method should provide insight into the mechanisms by which some heart arrhythmias are generated. In neurons, one could examine saltatory conduction and the effect of various degrees of demyelination, as in MS.

## Conclusion

As in the case of smooth muscle [18], we have been able to produce complete cardiac APs in PSpice simulation by placing a second BB (BB-2) into the Na^+ ^leg of the basic membrane units, which supplied an opposing current after a short time delay. This opposing current essentially deactivated the inward Na^+ ^current that was supplied by BB-1. Propagation in cardiac muscle occurred at physiological velocities when either no gj-channels or only one gj-channel was present. The propagation velocity became very fast and non-physiological when many gj-channels were present (e.g. 100 or 1000).

The Appendix presents a more complicated circuit for regulating the shape of the cardiac AP (e.g. to produce the typical spike and plateau of the cardiac action potential).

## Appendix

The circuit shown in Figure 5 depicts the novel circuit design for achieving a closer comparison to the physiological waveform for the cardiac action potential. In this circuit, BB-1 mimics the function of the fast Na^+ ^channel and BB-2 mimics the function of the slow Na^+ ^channel. Each leg contains a buffer amplifier (unit gain), a comparator (open-loop operational amplifier) and a delay element (RC time constant). The two BBs function in such a way that the sum of their output currents is equal to the instantaneous current at any time. The RC time delay element contained in each leg produces a corresponding delay time (i.e. the fast Na^+ ^channel leg has a shorter delay time and the slow Na^+ ^channel leg has a longer one). The buffer elements isolate both legs from BB-1 in order to avoid any hindrance in its function. The comparator is used to trigger the BBs with a fixed peak-to-peak voltage. The output of the comparator is connected to a voltage-divider network. The required voltage swing input for BB-1 and BB-2 is 110 mV. Since the comparator produces a rail-to-rail output voltage swing (-5 V to +5 V), the voltage-divider network circuit is used to scale down the output voltage swing from 10 V to 110 mV. An additional RC delay element was added at the inputs of BB-1 and BB-2 to smooth the edges of the AP. Using this method, one can include additional legs as needed to mimic the more ideal AP. The output waveform shown in Figure 6 was obtained by applying an I_stim _(current stimulus signal) at the inside surface of the cell.

**Table 2:** Black-box values (GTABLE) for surface membrane unit and junctional membrane unit.

**A. Surface membrane unit**			
**Black-box #1**		**Black-box #2**	

**Voltage (mV)**	**Current (nA)**	**Voltage (mV)**	**Current (nA)**

-80	0	-80	0
+30	-1.77	+30	+1.0

**B. Junctional membrane unit**			

**Black-box #1**		**Black-box #2**	

-80	0	-80	0
+30	-0.177	+30	+0.10

# We found that the two entries into the GTABLE are sufficient to obtain the waveform desired.

**Table 3:** Summary of TPT and θ values as a function of number of gap-junction channels.

**Number of gap-junction channels **^#^	**Rgj (MΩ)**	**TPT (ms)**	**Θ (cm/s)**
0	100,000	12.9	10.4
1	10,000	7.1	19.0
10	1,000	2.5	54.0
100	100	0.2	675
1,000	10	0.0004	337500

# Each gap-junction channel was assumed to have a conductance of 100 picosiemens (pS)
